# YiQiFuMai Powder Injection Protects against Ischemic Stroke via Inhibiting Neuronal Apoptosis and PKC*δ*/Drp1-Mediated Excessive Mitochondrial Fission

**DOI:** 10.1155/2017/1832093

**Published:** 2017-12-24

**Authors:** Yingqiong Xu, Yan Wang, Guangyun Wang, Xinyi Ye, Jiangwei Zhang, Guosheng Cao, Yazheng Zhao, Zhen Gao, Yuanyuan Zhang, Boyang Yu, Junping Kou

**Affiliations:** Department of Complex Prescription of TCM, State Key Laboratory of Natural Products, Jiangsu Key Laboratory of TCM Evaluation and Translational Research, China Pharmaceutical University, Nanjing, China

## Abstract

YiQiFuMai (YQFM) powder injection has been reported to be used in cardiovascular and nervous system diseases with marked efficacy. However, as a treatment against diseases characterized by hypoxia, lassitude, and asthenia, the effects and underlying mechanisms of YQFM in neuronal mitochondrial function and dynamics have not been fully elucidated. Here, we demonstrated that YQFM inhibited mitochondrial apoptosis and activation of dynamin-related protein 1 (Drp1) in cerebral ischemia-injured rats, producing a significant improvement in cerebral infarction and neurological score. YQFM also attenuated oxidative stress-induced mitochondrial dysfunction and apoptosis through increasing ATP level and mitochondria membrane potential (Δ*ψ*m), inhibiting ROS production, and regulating Bcl-2 family protein levels in primary cultured neurons. Moreover, YQFM inhibited excessive mitochondrial fission, Drp1 phosphorylation, and translocation from cytoplasm to mitochondria induced by oxidative stress. We provided the first evidence that YQFM inhibited the activation, association, and translocation of PKC*δ* and Drp1 upon oxidative stress. Taken together, we demonstrate that YQFM ameliorates ischemic stroke-induced neuronal apoptosis through inhibiting mitochondrial dysfunction and PKC*δ*/Drp1-mediated excessive mitochondrial fission. These findings not only put new insights into the unique neuroprotective properties of YQFM associated with the regulation of mitochondrial function but also expand our understanding of the underlying mechanisms of ischemic stroke.

## 1. Introduction

Ischemic stroke is a devastating cerebral vascular disease induced by insufficient cerebral blood flow, which is characterized by high mortality and morbidity [1]. A series of complex cellular and biochemical molecular mechanisms are involved in the pathology, such as the breakdown of cellular integrity and ionic homeostasis, oxidative stress, and release of excitotoxic glutamate, eventually leading to the loss of neurological functions and cell death [2]. Above all, the production of reactive oxygen species (ROS) is immediately increased after acute ischemic stroke and rapidly overwhelms antioxidants [3]. Oxidative stress-induced neuronal apoptosis and mitochondrial dysfunction are critical to the development of ischemic stroke [4]. Hydrogen peroxide (H_2_O_2_), one of the main reactive ROS, can cause lipid peroxidation, DNA damage, and mitochondria dysfunction and is thought to be the major precursor of free radicals [5]. Exogenous H_2_O_2_ has been reported to induce neuronal cell apoptosis in the central nervous system (CNS) and has been used to produce oxidative stress-induced apoptosis models [6]. Meanwhile, it has been also used in a model of cerebral ischemic in vitro [7, 8].

Mitochondria are essential cellular organelles in eukaryotic cells [9]. As the major source of ROS production, mitochondria are susceptible to oxidative damage [10]. Furthermore, mitochondria are highly dynamic organelles with frequent fission and fusion to maintain mitochondrial function, morphology, and subcellular distribution [11]. Neurons are particularly sensitive to changes in mitochondrial dynamics due to their high energy demands [12]. The balance between mitochondrial fission and fusion is highly relevant to the pathological processes of many CNS diseases, including acute stroke [13–15] and some neurodegenerative diseases [16]. In mammals, dynamin-related protein 1 (Drp1) is the main protein regulating mitochondrial fission and acting as an intrinsic factor in mitochondria-dependent apoptosis pathway [17, 18]. Drp1 is recruited to the mitochondrial membrane from cytosol and phosphorylated at ser-616, leading to mitochondrial fission. Inhibition of Drp1 by siRNA or Mdivi-1 (a specific inhibitor of Drp1) counteracts the conversion to a punctiform mitochondrial phenotype and attenuates insertion and oligomerization of Bax, thus blocking cell apoptosis, decreasing infarction and neurological deficits after transient focal ischemia [19]. Recent evidence indicates that free radical generation after ischemic and reperfusion injury causes mitochondrial damage mediated by mitochondrial translocation of PKC*δ* [20]. In addition, PKC*δ* can be activated and phosphorylate Drp1 at Ser-579/616 under oxidative stress, leading to mitochondrial fragmentation and mitochondrial dysfunction, which contribute to hypertension-induced brain injury [21].

YiQiFuMai (YQFM) powder injection is a modern lyophilized preparation based on a well-known traditional Chinese medicine formula Sheng Mai San, which is composed of three herbs: Radix Ginseng, Radix Ophiopogonis, and Fructus Schisandrae. Considerable clinical evidences have confirmed that YQFM has superior clinical efficacy and fewer side effects for treating chronic heart failure and hypertrophic obstructive cardiomyopathy [22–24]. Furthermore, YQFM was approved in 2007 by the China Food and Drug Administration for the therapeutic means of microcirculatory disturbance-related diseases [25]. The components of YQFM, for example, Rg1, Rb1, Rd, and schizandrin exerted a protective effect on cerebral ischemia-induced damage through the suppression of oxidative stress, inflammation, and mitochondria apoptosis pathway [26–31]. Among the components of YQFM, there are some of which have regulations on mitochondrial fission or PKC*δ*. For example, Re rescues methamphetamine-induced mitochondrial dysfunction, oxidative stress, and apoptosis in neuronal cells by inhibition of PKC*δ* [32, 33]. Rg5 prevents cardiomyocyte cell apoptosis with reduced Drp1 recruitment and mitochondrial fission [34]. Rg1 prevents myocardial hypoxia/reoxygenation injury by regulating mitochondrial dynamics imbalance [35]. Recent studies have also indicated that YQFM protects against ischemic injury through regulating endoplasmic reticulum stress-mediated neuronal damage [36]. YQFM also ameliorates blood-brain barrier (BBB) dysfunction through NF-*κ*B and ROCK1/MLC signaling pathways [37, 38]. More importantly, YQFM is effective in mitigating myocardial ischemia/reperfusion injury by modulating AMPK activation-mediated mitochondrial fission [39]. However, the effects and potential mechanisms of YQFM on ischemic stroke-induced neuronal mitochondrial fission have not been fully elucidated.

Considering the key roles of mitochondria-mediated neuronal apoptosis and the close links between mitochondrial dynamics with apoptosis in CNS diseases, the present study is designed to investigate the protective effect of YQFM on ischemia-induced neuronal apoptosis through mitochondrial fission pathway using cerebral ischemia-injured rats and primary cortical neurons. Our results confirm the efficacy of YQFM in ischemic stroke and provide novel insights into its mechanisms of cerebral ischemia treatment.

## 2. Materials and Methods

### 2.1. Materials

YQFM was purchased from Tasly Pharmaceutical Co. Ltd. (Tianjin, China; batch number 20121210). Neurobasal medium, B-27 supplement (50x, minus antioxidants), soybean trypsin inhibitor (SBTI), fetal bovine serum (FBS), L-glutamine, Alexa Fluor® 488 donkey anti-mouse antibody, Alexa Fluor 594 donkey anti-rabbit antibody, and MitoSOX™ red mitochondrial superoxide indicator were purchased from Thermo Fisher Scientific (San Jose, CA, USA). Mdivi-1, rottlerin, tetramethylrhodamine ethyl ester perchlorate (TMRE), DNase I, cytosine *β*-D-arabinofuranoside (AraC), poly-L-lysin (PLL), HEPES, N-acetyl-L-cysteine (NAC), and hydrogen peroxide (H_2_O_2_) were purchased from Sigma-Aldrich (St. Louis, MO, USA). MitoTracker® deep red FM was purchased from Life Technologies (CA, USA). Streptomycin, penicillin, 3-(4,5-dimethylthiazol-2-yl)-2,5-diphenyl tetrazoliumbromide (MTT), 2,3,5-triphenyltetra-zolium chloride (TTC), and bovine serum albumin (BSA) were purchased from AMRESCO (Solon, OH, USA). Caspase-3 activity kit, ATP assay kit, cell/tissue mitochondria isolation kit, 4′,6-diamidino-2-phenylindole (DAPI), and fluorescent kit for DCFH-DA were obtained from Beyotime Institute of Biotechnology (Shanghai, China). RIPA lysis buffer, protease inhibitor, enhanced chemiluminescence (ECL) reagent, and Annexin V/PI apoptosis detection kit were from Vazyme Biotech (Nanjing, China). Antibodies against Bax, Bak, Bcl-2, Bcl-xl, cleaved caspase-9, cleaved caspase-3, Drp1, p-Drp1 (Ser616), COXIV, and cytochrome C were purchased from Cell Signaling Technology (Danvers, MA, USA). Antibody against *β*-actin was from Bioworld Technology (Louis Park, MN, USA). PKC*δ* antibody, protein A/G PLUS agarose, and normal rabbit IgG were from Santa Cruz Biotechnology (Santa Cruz, CA, USA). ATP5A1 antibody was from Proteintech Group (Chicago, IL, USA). HRP-conjugated secondary antibodies were from Boster (Wuhan, China). Dimethyl sulfoxide (DMSO) was obtained from SunShine Bio (Nanjing, China).

### 2.2. Transit Focal Cerebral Ischemia/Reperfusion (I/R)

3-month-old male Sprague-Dawley rats weighing 280–300 g were purchased from the Laboratory Animal Center of Nanjing Qinglongshan (Nanjing, China). All animal experiments in this study were approved by Institutional Animal Care and Use Committee of China Pharmaceutical University. Experimental ischemic stroke model was induced in Sprague-Dawley rats by transit middle cerebral artery occlusion (tMCAO) and reperfusion. Rats were anesthetized with 4% chloral hydrate (0.1 ml/10 g) intraperitoneally. Neck vessels were exposed through a midline incision. The external carotid was ligated, and the common carotid artery was blocked with an artery clip. A 3-0 silicone-coated monofilament nylon suture was carefully inserted into the internal carotid artery until light resistance was encountered. After 90 min transient cerebral ischemia, the suture was withdrawn followed by 24 h reperfusion. The same treatment was carried out in sham group except that the suture was not advanced into the internal carotid artery. Body temperature was maintained at 37.0 ± 0.5°C during surgery using a temperature-controlled heating pad. The animals were given free access to water and food until sampling. The rats were sacrificed after YQFM administration for 24 h and followed by subsequent experiments.

### 2.3. Experimental Groups

YQFM powder was dissolved in 0.9% sodium chloride. To confirm the effects of YQFM on cerebral I/R induced rats, the animals were randomly divided into 4 groups (*n* = 8 for each group): (1) sham; (2) sham + YQFM: YQFM was injected intraperitoneally in rats at the dose of 0.957 g/kg; (3) I/R group: rats were subjected to 1.5 h ischemia followed by 24 h reperfusion; and (4) I/R + YQFM: YQFM (0.957 g/kg) was injected intraperitoneally after 1.5 h ischemia.

### 2.4. Assessment of Cerebral Infarct Size

Brains were rapidly removed after reperfusion and mildly frozen to keep the morphology intact during slicing. Brains were sliced into 5 serial coronal sections with the aid of brain matrices. Sections were placed into 2% 2,3,5-triphenyltetra-zolium chloride (TTC) at 37°C for 10 min and then transferred into 4% paraformaldehyde for fixation at 4°C overnight. TTC-stained sections were collected by a Canon EOS D60 digital camera. The infarct area was measured using ImageJ software (NIH, Bethesda, MD, USA).

### 2.5. Neurological Deficit Scoring

Neurological deficits of rats in different groups were examined after 24 h of reperfusion (*n* = 8) on a scoring system: (0) no deficits; (1) unable to extend the left forepaw fully; (2) circling to the left; (3) falling to the left; (4) no spontaneous walking and reduced consciousness; and (5) dead [40]. The neurological scoring test was carried out by one of the coauthors blinded to the experimental groups.

### 2.6. Hematoxylin and Eosin (H&E) Staining

Histomorphological analysis was measured by H&E staining. Brains were immediately removed after reperfusion and fixed with 4% paraformaldehyde for 24 h. 3 *μ*m sections were cut from the coronal plane of the paraffin-embedded tissue. The sections were dehydrated stepwise using alcohol and stained with H&E.

### 2.7. Immunofluorescence Staining and Quantification of Colocalization

For the brain tissues, 3 *μ*m paraffin sections were deparaffinized in xylene for 30 min, incubated in absolute ethanol for 10 min, and rehydrated via graded concentrations of alcohol. Endogenous peroxidase activity was quenched in a solution containing 10% methanol plus 3% H_2_O_2_ in PBS for 30 min. Antigen retrieval was performed by heating the samples at 95°C in 0.01 M citrate buffer (pH 6.0), after which sections were washed with PBS. The sections were then permeabilized with 0.1% Triton X-100, incubated with 5% bovine serum albumin (BSA) for 1.5 h to block nonspecific staining, and then incubated with primary antibodies overnight at 4°C in 5% BSA. After several washings, sections were incubated in fluorochrome-coupled secondary antibody for 2 h and the nuclei were stained with DAPI for 5 min. After being rinsed with PBS, the sections were visualized under a confocal scanning microscope (CLSM, LSM700, Zeiss, Germany). Quantitative colocalization of ATP5A1 with Drp1 was performed using the Image-Pro Plus 6.0 software (Media Cybernetics, Bethesda, MD, USA), which provides Manders' coefficients for the overlap of the images according to previous study [41]. Its values range between 0 and 1.0. A value of 1 represents strong positive correlation and 0 indicates that there is no discernable correlation. All localization images are analyzed with single plane images.

### 2.8. Primary Cultured Cortical Neurons

Primary cultures of cortical neurons were prepared from embryonic day 16–18 Sprague-Dawley rats according to previously described methods [42]. Briefly, the cerebral cortices were dissected out and the meninges were carefully removed under a microscope. Neocortices were then minced by trituration with a Pasteur pipette and digested in trypsin. Dispersed cells were diluted to a concentration of 1 × 10^6^ cells/ml and plated on poly-L-lysine coated plates in neurobasal medium, containing 5% fetal bovine serum (FBS), 10 U/ml penicillin, 10 *μ*g/ml streptomycin, 2% B27 Supplement Minus AO, 10 mM HEPES, and 0.5 mM glutamine at 37°C with 5% CO_2_. AraC was added to the medium after 24 h to inhibit glial and nonneuronal cell growth. Half of the medium was changed every two days. The cells were used for experiments between 6 and 9 days in vitro.

### 2.9. Cell Viability Assay

The cell viability was measured by MTT assay. The cortical neurons were plated in 96-well plates and grown in neurobasal medium for 6 days. After treatment, 100 *μ*l of MTT (5 mg/ml) was added to each well and incubated at 37°C for 4 h. The medium was then aspirated and 150 *μ*l of DMSO was added to each well to dissolve formazan crystals with shaking for 10 min. The optical density (OD) values were measured by a microplate reader (Epoch, BioTek, USA) at a detective wavelength of 570 nm, with a reference wavelength of 650 nm. Cell viability was expressed as a percentage with the control group, which was taken as 100%.

### 2.10. Caspase-3 Activity Assay

The caspase-3 activity was determined using a caspase-3 activity kit (Beyotime Biotech, Haimen, China). Treated cells were lysed and centrifuged. The supernatant was incubated with 2 mM of Ac-DEVD-pNA substrate at 37°C for 4 h in 96-well plates. The absorbance was detected by a microplate reader (Epoch, BioTek, USA) at 405 nm. The protein levels were measured by the Bradford method (Beyotime Biotech, Haimen, China). The caspase-3 activity was normalized to the protein concentration of each group.

### 2.11. Analysis of Cell Apoptosis with Flow Cytometry

Primary cortical neurons were harvested and resuspended into a single cell solution of 1 × 10^6^ cell/ml. Cells were then stained using the FITC-Annexin V/PI apoptosis detection kit (Vazyme Biotech, Nanjing, China) according to the manufacturer's instruction. Samples were then detected using FACSCalibur flow cytometer (BD Biosciences, San Diego, CA, USA). Data were analyzed using FlowJo software (TreeStar Inc., Ashland, OR, USA).

### 2.12. Measurement of ATP Levels

Adenosine 5′-triphosphate (ATP) levels were performed using the ATP assay kit (Beyotime, Shanghai, China) following the manufacturer's instructions. Briefly, treated cells were harvested and lysed. After centrifugation, 50 *μ*l supernatant and 50 *μ*l ATP detection buffer were mixed together in the dark. Luminescence was measured using LUMIstar Omega plate reader (BMG LABTECH GmbH, Germany). The concentration of ATP was normalized to the protein concentration of each group, which was determined by the Bradford method.

### 2.13. Mitochondrial Membrane Potential (Δ*ψ*m)

Mitochondrial membrane potential was detected using fluorescent dye TMRE staining. In brief, cells were washed with PBS and then incubated with TMRE staining solution (final concentration of 500 nM) for 30 min at 37°C. The cells were then washed twice with PBS and fluorescence, was read using a fluorescent plate reader (Thermo Scientific Varioskan® Flash, USA) with the excitation wavelength of 540 nm and emission wavelength of 595 nm. Data were shown as TMRE fluorescence intensity.

### 2.14. Reactive Oxygen Species (ROS)

The mitochondrial ROS level was detected using MitoSOX red mitochondrial superoxide indicator, which penetrates live cells and selectively localizes to the mitochondria. According to the manufacturer's instructions, cells were rinsed twice with PBS and then stained with 5 *μ*M MitoSOX red diluted in PBS for 30 min at 37°C in the dark. Subsequently, the amount of intracellular ROS was measured using a fluorescent plate reader with the excitation of 510 nm and emission of 580 nm.

For intracellular ROS production, cells were washed twice with PBS, and then incubated with ROS specific fluorescent probe dye DCFH-DA for 30 min at 37°C. After washing, the DCF fluorescence was observed using a fluorescence microscope (Olympus Corporation, Japan) at 40x magnification.

### 2.15. Mitochondrial Fission and Drp1 Translocation Analysis

For mitochondrial fission assay, primary cortical neurons were washed with PBS and then incubated with 400 nmol/l MitoTracker Deep Red FM for 30 min at 37°C in the dark. The structure of mitochondria was then observed using confocal microscopy.

For Drp1 translocation assay, neurons were washed with PBS and incubated with 400 nmol/l MitoTracker Deep Red FM for 30 min at 37°C in the dark. The cells were then fixed, permeabilized, and incubated with anti-Drp1 antibody, followed by incubation with Alexa Fluor 488 conjugated donkey anti-mouse antibody. The structure of mitochondria and translocation of Drp1 were viewed using confocal microscopy. The quantification of colocalization of Drp1 with MitoTracker was analyzed by Image-Pro Plus 6.0 software as mentioned above.

### 2.16. Western Blot Analysis

The cortical neurons were lysed with RIPA buffer supplied with protease inhibitor cocktail. For assay in brain tissues obtained from the cortical margin of infarct areas, the tissues were homogenized in RIPA buffer supplied with protease inhibitor cocktail. Proteins were centrifuged at 12000 rpm for 10 min at 4°C. The extracted proteins were separated by 12.5% SDS-PAGE and transferred to polyvinylidene fluoride (PVDF) membranes (Millipore Corporation, Billerica, MA, USA). The membranes were blocked and incubated with various primary antibodies at 4°C overnight. Membranes were then probed with peroxidase conjugated secondary antibody. After washing, the antigen-antibody complexes were detected with ECL (Vazyme Biotech, Nanjing, China) and visualized using ChemiDoc™ MP System (Bio-Rad, Hercules, CA, USA). Results were quantified using the Image Lab™ software (version 4.1, Bio-Rad, Hercules, CA, USA).

For the mitochondrial protein analysis, mitochondrial fractions were isolated from primary cortical neurons or brain issues with the mitochondria isolation kit (Beyotime, Shanghai, China) according to the manufacturer's protocol. Then, the mitochondrial and cytosolic fractions proteins were determined as described above.

### 2.17. Immunoprecipitation

The protein A/G-PLUS agarose beads were washed with RIPA lysis buffer three times and then incubated with 2 *μ*g indicated antibodies at 4°C overnight on a rocker table. Mitochondrial or cytosolic fractions of rat brain or cultured cell homogenates were incubated with prepared agarose-antibody complex for 4 h at 4°C. 30 *μ*l of 2x SDS loading buffer was added to the washed agarose complex and then the mixture was boiled. The immunoprecipitates were separated on 12.5% SDS-PAGE and probed with the indicated antibodies.

### 2.18. Statistical Analysis

Statistical analysis of the data was performed using one-way analysis of variance (ANOVA) followed by Dunnett's post hoc test for multiple comparisons using GraphPad Prism 6.0 (La Jolla, CA, USA). Data were expressed as mean ± SD. *P* < 0.05 was considered significant.

## 3. Results

### 3.1. YQFM Ameliorates Cerebral I/R Injury Linked with Mitochondrial Apoptosis

**Figure 1:**
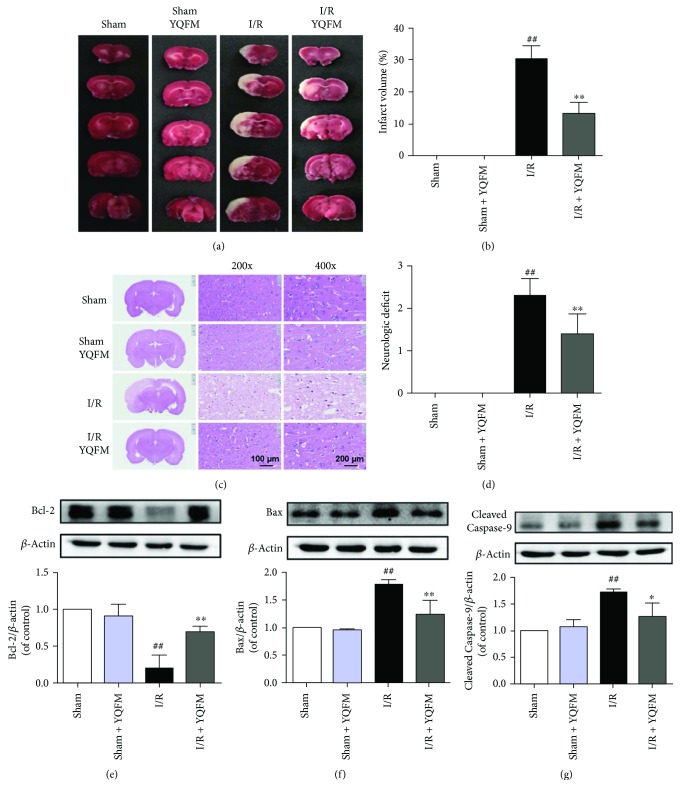
YQFM protects against cerebral I/R injury and mitochondrial apoptosis. Rats were subjected to 1.5 h of ischemia, followed by 24 h of reperfusion. YQFM (0.957 g/kg) was administered intraperitoneally after ischemia. (a) Representative TTC-stained brain sections. (b) Quantitative analysis of infarct volume. (c) H&E stained sections of rat brains. (d) Quantitatification of neurologic deficit scores (*n* = 8). Expression of Bcl-2 (e), Bax (f), and cleaved caspase-9 (g) in ischemic brain tissue. Results were expressed as mean ± SD from three independent experiments. ^##^*P* < 0.01 versus Sham, ^∗^*P* < 0.05 versus I/R, ^∗∗^*P* < 0.01 versus I/R.

We first investigated the effects of YQFM on rat cerebral I/R injury. We selected the dose (0.957 g/kg) from previous studies performed in mice but adjusted to rats [38]. According to the body surface area conversion, the dose of mice is about 1.4 times to rats; thus, 0.957 g/kg of YQFM was used in rats in our study. Rats were subjected to 90 min of ischemia and 24 h of reperfusion. YQFM (0.957 g/kg) was administered intraperitoneally after 90 min of ischemia. The TTC-staining image and quantitative analysis of brain infarction demonstrated that YQFM treatment significantly reduced infarct size after cerebral I/R injury (Figures 1(a) and 1(b)). H&E staining showed that cerebral I/R induced cell loss and numerous vacuolated spaces compared with sham group. While treatment with YQFM ameliorated such histopathological damage by decreasing cell loss and vacuolation. YQFM alone did not influence the morphologic features of brains (Figure 1(c)). Administration of YQFM also resulted in significant improvement of neurobehavioral deficits compared with I/R group (Figure 1(d)). Furthermore, YQFM notably increased the expression of Bcl-2 and decreased the expression of Bax and cleaved caspase-9 after cerebral I/R injury. No obvious changes were observed in the group treated with YQFM alone (Figures 1(e)–1(g)). The protective effect of YQFM in I/R-injured rats and apoptosis-related factors expression were similar to those in mice in previous study, which indicated a stable efficiency of YQFM. Overall, these results indicated that YQFM protects against cerebral ischemic injury linked with mitochondrial apoptosis pathway.

### 3.2. YQFM Suppresses Cerebral I/R-Induced Drp1 Activation

**Figure 2:**
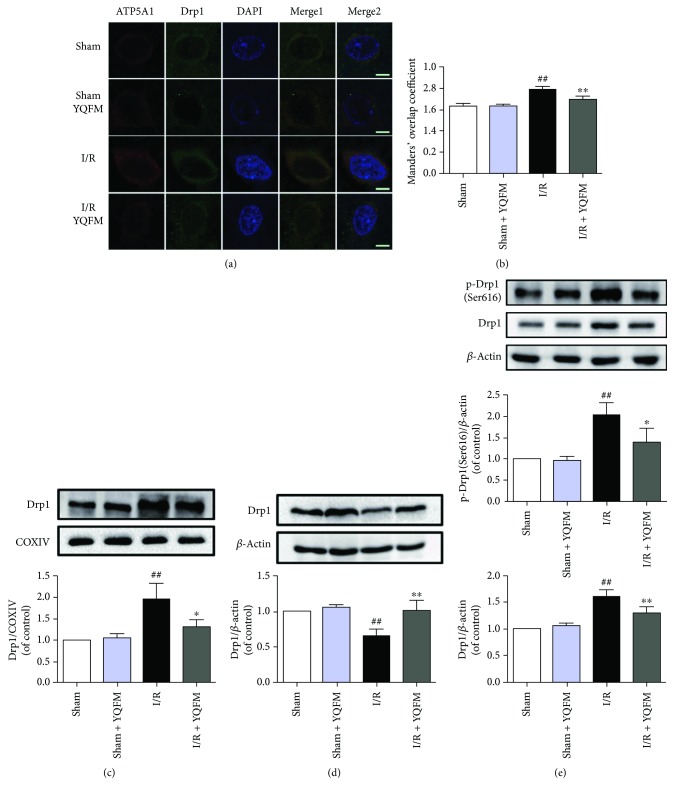
YQFM suppresses translocation, expression and phosphorylation of Drp1 in I/R-injured rats. (a) Immunofluorescent staining of mitochondria and Drp1 in the cerebral cortex of ischemic hemisphere. Mitochondria were marked with ATP5A1 antibodies and nuclei were stained with DAPI. Bar = 5 *μ*m. (b) The colocalization of ATP5A1 with Drp1 was assessed on the basis of Manders' overlap coefficients. Expression of Drp1 in the mitochondrial (c) and cytosolic (d) fractions. (e) Total levels of Drp1 and p-Drp1 (Ser616) in rat cerebral cortex of ischemic hemisphere. Results were expressed as mean ± SD from three independent experiments. ^##^*P* < 0.01 versus Sham, ^∗^*P* < 0.05 versus I/R, and ^∗∗^*P* < 0.01 versus I/R.

As fragmentation of mitochondria participates in the process of apoptosis [17], we further evaluated the effect of YQFM on mitochondrial fission after cerebral I/R injury. Drp1, a vital mitochondrial fission protein, regulates mitochondrial morphology by promoting fission [43]. The immunofluorescent results and quantitative analysis by Manders' overlap coefficients showed that cerebral I/R induced translocation of Drp1 from cytoplasm to mitochondria, which was reduced by YQFM treatment, manifesting the inhibition of Drp1 recruitment (Figures 2(a) and 2(b)). Consistently, Western blot analysis showed that the level of Drp1 was increased in the mitochondrial fractions and decreased in the cytosolic fractions in the cerebral cortex of ischemic hemisphere. While the translocation of Drp1 was inhibited in response to YQFM treatment (Figures 2(c) and 2(d)). Meanwhile, YQFM also decreased Drp1 phosphorylation at Ser616 and total Drp1 expression induced by cerebral I/R (Figure 2(e)). As a result, YQFM prevented I/R-induced mitochondrial fission through inhibiting Drp1 activation and translocation in the cerebral cortex.

### 3.3. YQFM Protects Primary Cortical Neurons against H_2_O_2_-Induced Apoptosis

**Figure 3:**
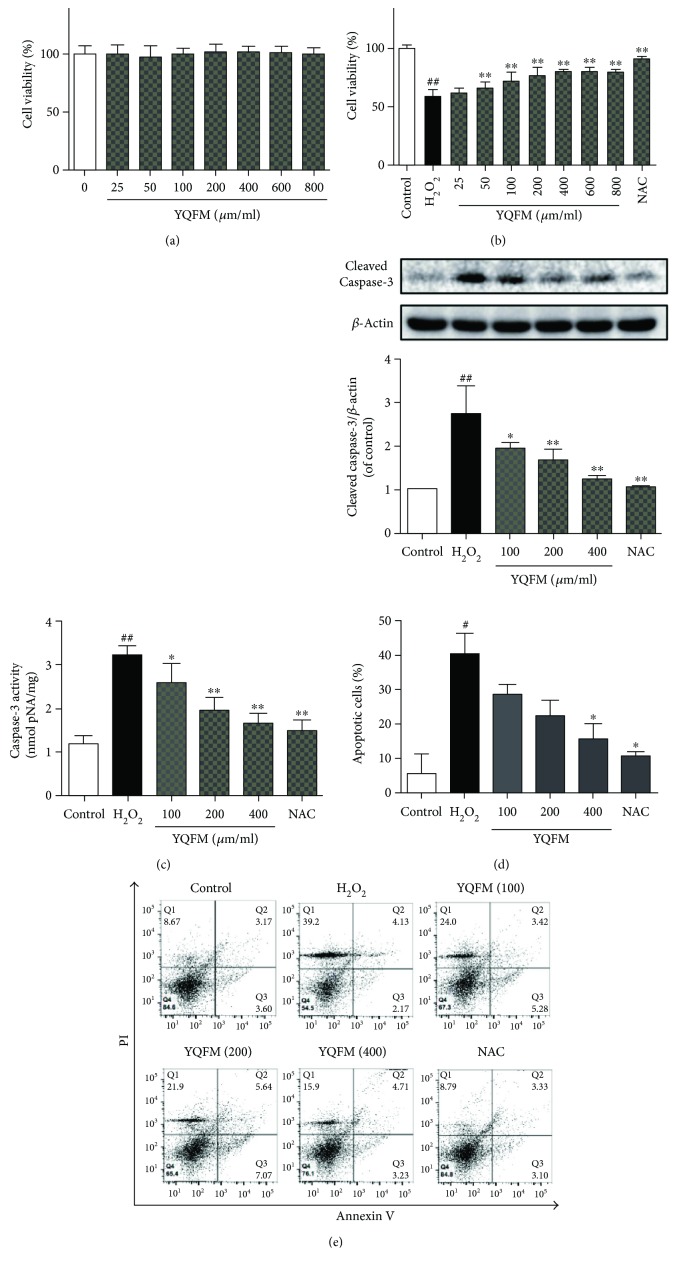
YQFM protects primary cortical neurons from H_2_O_2_-induced apoptosis. (a) Neurons were treated with different concentrations of YQFM (25–800 μg/ml) for 24 h. Cell viability was measured using MTT assay. (b) Neurons were pretreated with YQFM (25–800 μg/ml) or NAC (500 μM) for 6 h before addition of H_2_O_2_ (100 *μ*M) for 12 h. Cell viability was measured using MTT assay. Neurons were pretreated with YQFM (100, 200, and 400 *μ*g/ml) or NAC (500 *μ*M) for 6 h before exposure of H_2_O_2_ (100 *μ*M) for 12 h. (c) Caspase-3 activity was assayed using the caspase-3 activity assay kit. (d) Protein level of cleaved caspase-3 was detected by Western blot. (e) Flow cytometric analysis of Annexin V-FITC/PI stained neurons and quantification as the percentage of apoptotic cells. Results were expressed as mean ± SD from three independent experiments. ^#^*P* < 0.05 versus control, ^##^*P* < 0.01 versus control, ^∗^*P* < 0.5 versus H_2_O_2_, ^∗∗^*P* < 0.01 versus H_2_O_2_.

Mitochondrial dysfunction has been reported to lead to oxidative stress [44] and is also thought to play a critical role in the pathological process of ischemic attack [3]. To further determine the mechanisms of YQFM on neuronal protection, we investigated the effects of YQFM on oxidative stress-injured primary cortical neurons in vitro. We first determined the cytotoxicity of YQFM on untreated primary cortical neurons. Treatment with YQFM for 24 h at the concentrations from 25 to 800 *μ*g/ml had no effect on the viability of cortical neurons, suggesting that YQFM had no toxicity or inhibition on the growth of cortical neurons (Figure 3(a)). Then, cells were pretreated with various concentrations of YQFM (25–800 *μ*g/ml) for 6 h before the addition of H_2_O_2_ (100 *μ*M) for 12 h. Cells pretreated with 500 *μ*M NAC served as positive control. MTT assay demonstrated that H_2_O_2_-induced reduction of neuronal viability was significantly reversed by the pretreatment of YQFM with increasing dosage (100–800 *μ*g/ml) (Figure 3(b)). We then investigated if YQFM could inhibit H_2_O_2_-induced apoptosis. Three indices were applied to evaluate the protective effects of YQFM: (1) caspase-3 activity determined by a caspase-3 activity assay kit; (2) protein expression of cleaved caspase-3 detected by Western blot; (3) Annexin V/PI staining analyzed by flow cytometry. As shown in Figures 3(c) and 3(d), the activity of caspase-3 and expression of cleaved caspase-3 were remarkably increased by H_2_O_2_ treatment in neurons, which was significantly reversed with YQFM incubation at concentrations of 100–400 *μ*g/ml. Consistent with the inhibition of caspase-3 activation, flow cytometry results showed that pretreatment of YQFM or NAC significantly reduced apoptotic cells induced by H_2_O_2_ exposure (Figure 3(e)). These results demonstrated that YQFM inhibits neuronal apoptosis induced by oxidative stress.

### 3.4. YQFM Inhibits H_2_O_2_-Induced Neuronal Apoptosis via Ameliorating Mitochondrial Dysfunction and Apoptosis

**Figure 4:**
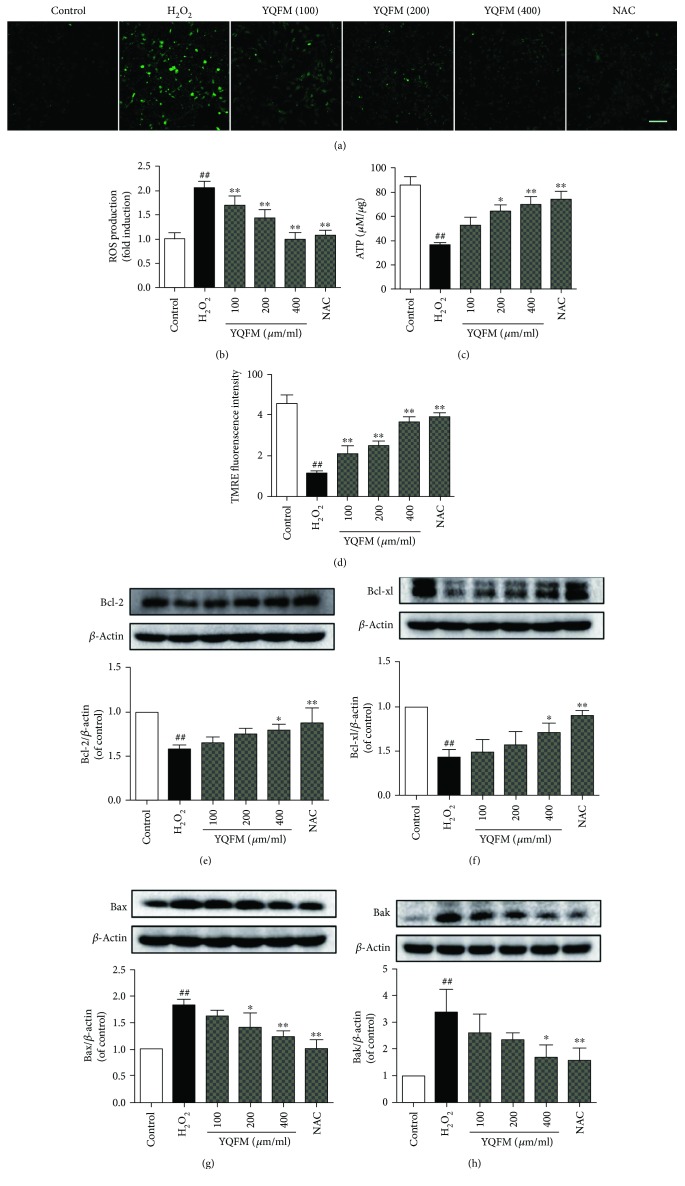
YQFM protects against H_2_O_2_-induced mitochondrial dysfunction and apoptosis. Neurons were pretreated with YQFM (100, 200, and 400 *μ*g/ml) or NAC (500 *μ*M) for 6 h before exposure of H_2_O_2_ (100 *μ*M) for 12 h. (a) Intracellular ROS level was detected in neurons stained with DCFH-DA. Bar = 100 *μ*m. (b) Mitochondria-derived ROS generation was determined using a mitochondrial superoxide anion specific fluorescent probe (MitoSOX red). The fluorescence was measured using a fluorometer. (c) Cellular ATP content was measured by an ATP assay kit. (d) Mitochondrial membrane potential was evaluated by TMRE using a fluorometer. Protein expression of Bcl-2 (e), Bcl-xl (f), Bax (g), and Bak (h) was detected by Western blot. Data were presented as mean ± SD from three independent experiments. ^##^*P* < 0.01 versus control, ^∗^*P* < 0.5 versus H_2_O_2_, ^∗∗^*P* < 0.01 versus H_2_O_2_.

We then investigated the influence of YQFM on mitochondria-dependent neuronal apoptosis and mitochondrial dysfunction. As mitochondria are the major source of ROS production, we observed the effects of YQFM on intracellular and mitochondrial ROS generation. As illustrated in Figure 4(a), pretreatment of neurons with YQFM attenuated the increase in intracellular ROS induced by H_2_O_2_. YQFM also effectively decreased mitochondrial ROS level in neurons stained with a mitochondrial superoxide anion-specific fluorescent probe (Figure 4(b)). We then determined mitochondrial function by assaying ATP production and mitochondria membrane potential (Δ*ψ*m). Consistently, H_2_O_2_ induced decrease of ATP production and collapse of Δ*ψ*m, which were reversed by YQFM treatment (Figures 4(c) and 4(d)). Mitochondrial depolarization was confirmed by using FCCP, a protonophore and uncoupler of mitochondrial oxidative phosphorylation. Cells immediately decreased their TMRE fluorescence upon addition of FCCP (10 *μ*M) (data not shown). Furthermore, we studied the effects of YQFM on expression of protein related with mitochondrial apoptosis. As shown in Figures 4(e)–4(h), the expression of Bcl-2 and Bcl-xl was decreased, and the expression of Bax and Bak was significantly increased upon H_2_O_2_ induction in neurons, which could be remarkably reversed by pretreatment with YQFM. Thus, YQFM exerts neuronal protection via regulating mitochondrial function and ameliorating apoptosis.

### 3.5. YQFM Attenuates Excessive Mitochondrial Fission in Neurons Injured by H_2_O_2_

**Figure 5:**
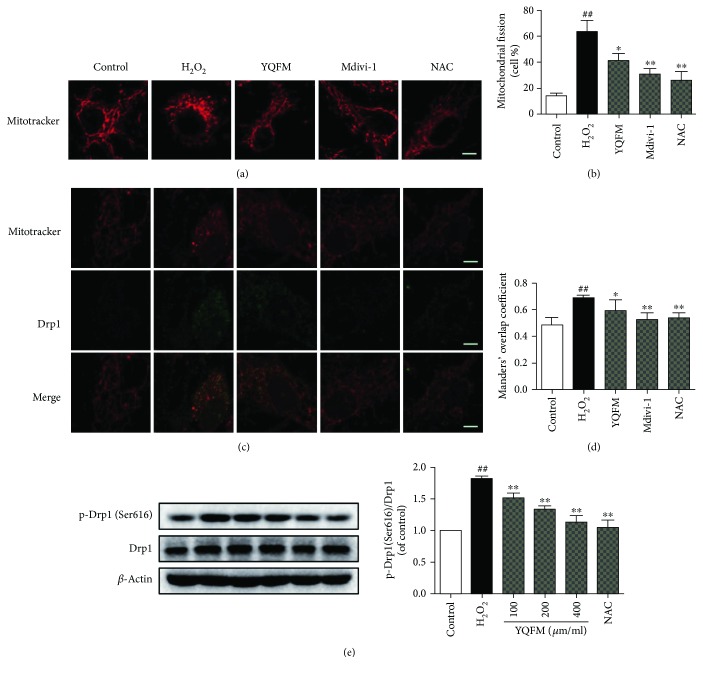
YQFM attenuates H_2_O_2_-induced excessive mitochondrial fission in neurons. Neurons were pretreated with YQFM (400 *μ*g/ml), mdivi-1 (25 *μ*M), or NAC (500 *μ*M) for 6 h before exposure of H_2_O_2_ (100 *μ*M) for 6 h. (a) Mitochondrial morphology was viewed by MitoTracker Deep Red FM using confocal microscopy. Bar = 5 *μ*m. (b) Quantitative analysis of percentage of fragmented mitochondria. (c) Mitochondrial localization of Drp1 (green) and mitochondrial morphology (red) were imaged using confocal microscopy. Bar = 5 *μ*m. (d) Colocalization of Drp1 and MitoTracker was quantified on the basis of Manders' overlap coefficients. (e) Expression of Drp1 and p-Drp1 (Ser616) were detected by Western blot. Data were presented as mean ± SD from three independent experiments. ^##^*P* < 0.01 versus control, ^∗^*P* < 0.5 versus H_2_O_2_, ^∗∗^*P* < 0.01 versus H_2_O_2_.

Mitochondria are organized in a highly dynamic tubular network [11]. Cells bearing oxidative stress are subjected to excessive mitochondrial fission [44]. We visualized morphology of mitochondria upon H_2_O_2_ injury using MitoTracker Deep Red FM staining. Confocal imaging showed that the majority of mitochondria shifted from the normally elongated tubular structures into punctuated structures, when neurons were exposed to H_2_O_2_. Both YQFM and Drp1 inhibitor Mdivi-1 pretreatment efficiently prevented the punctuation and reserved the elongated morphology of mitochondria and reduced the rates of mitochondria fission in cortical neurons (Figures 5(a) and 5(b)). As shown in Figures 5(c) and 5(d), YQFM and Mdivi-1 also reduced the location of Drp1 at mitochondria, which was consistent with the effect of Mdivi-1. Moreover, phosphorylation of Drp1 at Ser616 was reduced by YQFM treatment at concentrations ranging from 100 to 400 *μ*g/ml in neurons exposed to H_2_O_2_ injury (Figure 5(e)). Therefore, YQFM attenuates excessive mitochondrial fission through regulating Drp1 activation in neurons injured by H_2_O_2._

### 3.6. YQFM Protects against H_2_O_2_-Induced Mitochondrial Fission through Inhibiting PKC*δ*-Mediated Drp1 Activation

**Figure 6:**
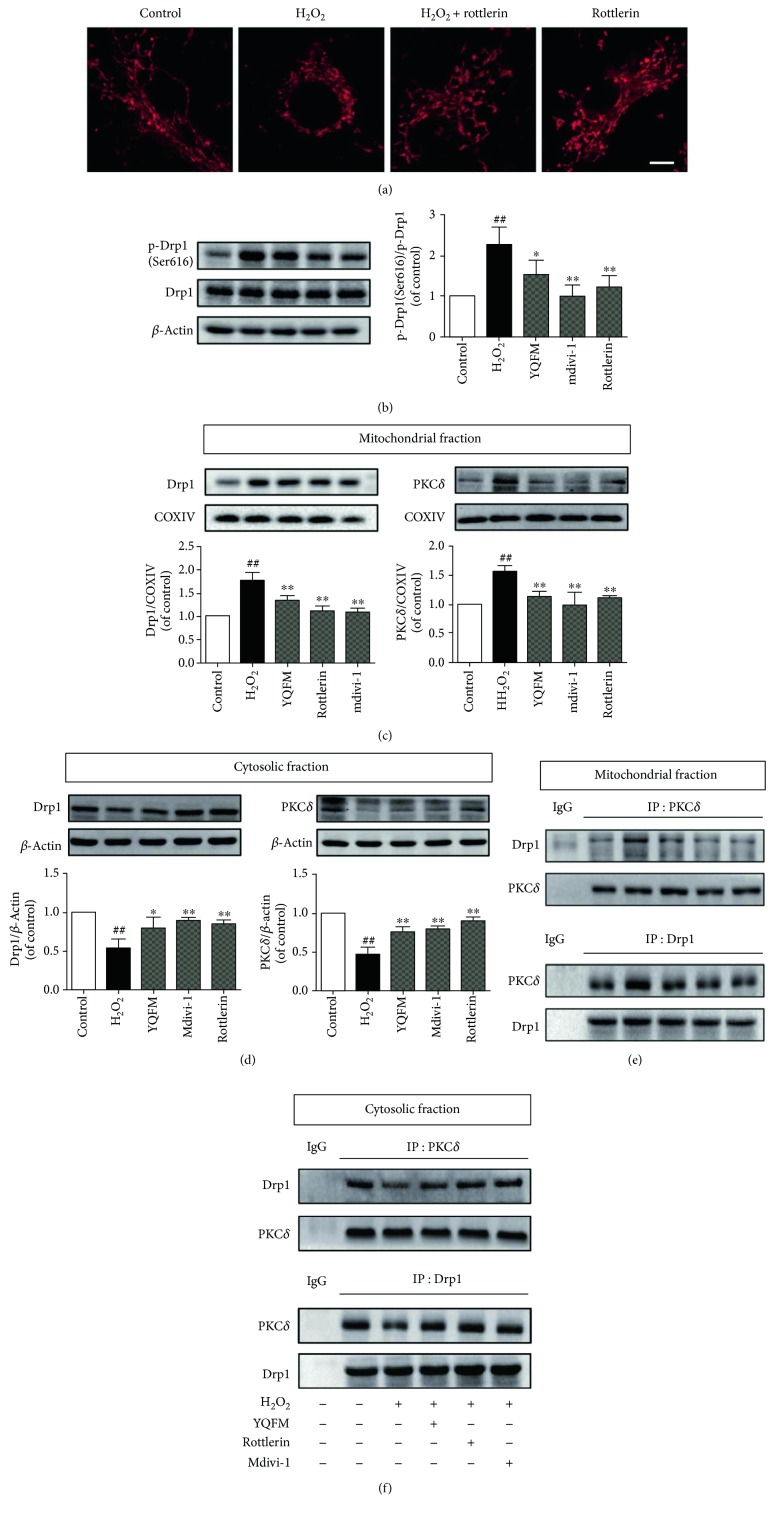
YQFM regulates mitochondrial fission through inhibition of PKC*δ*-mediated Drp1 activation. Neurons were pretreated with YQFM (400 *μ*g/ml), Rottlerin (1 *μ*M) or Mdivi-1 (25 *μ*M) for 6 h and then incubated with H_2_O_2_ (100 *μ*M) for 6 h. (a) Mitochondrial morphology stained with MitoTracker Deep Red FM. Bar = 5 *μ*m. (b) Protein expression of Drp1 and p-Drp1 (Ser616). Expression of Drp1 and PKC*δ* in the mitochondrial (c) or cytosolic (d) fraction was determined using Western blot. Coimmunoprecipitation of Drp1 and PKC*δ* in the mitochondrial (e) or cytosolic (f) fraction was detected by Western blot with indicated antibodies. Cytosolic or mitochondrial fractions were subjected to coimmunoprecipitation with anti-PKC*δ* antibody or anti-Drp1 antibody, and then the precipitates were analyzed by immunoblotting with anti-PKC*δ* and anti-Drp1 antibodies. IgG was loaded as a negative control. IB: immunoblotting, IP: immunoprecipitation and IgG: immunoglobulin G. ^##^*P* < 0.01 versus control, ^∗^*P* < 0.5 versus H_2_O_2_, ^∗∗^*P* < 0.01 versus H_2_O_2_.

PKC*δ* has been reported to interact with Drp1 and participate in the process of mitochondrial fission [21, 45, 46]. However, the effects of PKC*δ* on oxidative stress-induced mitochondrial fission in neuronal cells have not been investigated; thus, we identified the effect of rottlerin, a specific inhibitor of PKC*δ*, to demonstrate the involvement of PKC*δ*. As showed in Figure 6(a), inhibition of PKC*δ* by rottlerin suppressed H_2_O_2_-induced mitochondrial fission, while it had no influence on mitochondrial morphology of untreated neurons. Rottlerin also inhibited Drp1 phosphorylation (Ser616), which was similar to the effects of YQFM (Figure 6(b)). Translocation of PKC*δ* to mitochondria is one of the manifestations of its activation and function [47, 48]. YQFM, rottlerin as well as Mdivi-1 decreased the location of both PKC*δ* and Drp1 at mitochondria (Figure 6(c)). Accordingly, the cytosolic localization of PKC*δ* and Drp1 was increased by YQFM, rottlerin, and Mdivi-1 treatment (Figure 6(d)). We next investigated whether PKC*δ* could associate with Drp1 and regulate its localization. As shown in Figures 6(e) and 6(f), PKC*δ* and Drp1 coimmunoprecipitated in both mitochondrial and cytosolic fractions under normal conditions. However, the interaction of PKC*δ* and Drp1 was increased in mitochondria and decreased in cytoplasm when exposed to H_2_O_2_, suggesting the complex translocated to mitochondria under oxidative stress. YQFM pretreatment inhibited the interaction and translocation of Drp1 and PKC*δ* from cytoplasm to mitochondria, the effects of which were paralleled with rottlerin and Mdivi-1. These results demonstrated that the mechanism of YQFM on mitochondrial fission is mediated through the association and translocation of PKC*δ* and Drp1 in oxidative stress-induced neuronal apoptosis.

## 4. Discussion

In the present study, we explored the novel mechanisms of YQFM against neuronal injury through inhibiting PKC*δ*/Drp1-mediated excessive mitochondrial fission in ischemic stroke-injured rat and oxidative stress-induced primary cultured neurons. YQFM also attenuated mitochondrial dysfunction and apoptosis by regulating ATP level, Δ*ψ*m, ROS production, and Bcl-2 family proteins level. All together, our data demonstrated the protective effects of YQFM on ischemic stroke.

Mitochondria are key organelles in neuronal cells, which play important roles such as energy generation, calcium signaling, and apoptotic signaling [9]. Aberrant mitochondrial dynamics play critical roles in the pathological processes of ischemic stroke [14]. Previous studies have shown that YQFM protects against myocardial ischemia/reperfusion injury via AMPK pathway mediated mitochondrial fission [39] and oxygen-glucose deprivation-induced PC12 cell apoptosis through ER stress [36]. However, the effects of YQFM against mitochondrial dysfunction and excessive fission induced by ischemic stroke have not been fully elucidated. Our results showed that YQFM significantly attenuated oxidative stress-induced mitochondria apoptosis and damage by decreasing ROS production, increasing ATP level and Δ*ψ*m. YQFM also inhibited the expression, phosphorylation, and translocation of Drp1 in oxidative stress-induced primary neurons and cerebral ischemia-injured rats, producing a significant improvement in cerebral infarction and neurological score. Drp1 has been reported to play important roles in neuronal apoptosis or death in a number of CNS diseases [49]. The increased Drp1 phosphorylation at Ser616 activates and translocates Drp1 to the constriction sites of mitochondria, leading to mitochondrial fission [45, 50]. Inhibition of Drp1 using Mdivi-1 has been reported to exert neuroprotective effects against nerve injury after cerebral ischemia/reperfusion by the prevention of mitochondrial fission and apoptosis [51]. In our study, the inhibition of YQFM on Drp1 activation and mitochondrial fission was similar with the effects of Mdivi-1 treatment. Thus, YQFM attenuates neuronal damage induced by ischemic stroke through inhibiting Drp1-dependent mitochondrial fission. It has been reported that mitochondria form specific reticular or branched networks in the cytosol, which interact intimately in a dynamic way at some particular locations, comparable to ER network [52]. Meanwhile, inhibition of mitochondria fission by regulation of Drp1 could attenuate cell injuries from ER stress and ROS [53]. Thus, it implied that the regulation of YQFM in mitochondria fission may be linked to its modulation of ER stress as shown in the previous study. The specific relationship of its role in ER stress and mitochondria fission will be further explored in our following research.

As a dynamic protein, how is Drp1 activated and recruited to the mitochondrial outer membrane to mediate fission? Previous studies have suggested that PKC*δ* is activated immediately after the onset of ischemia reperfusion and that excessive ROS also induces PKC*δ* activation [54]. Drp1 phosphorylation can be attenuated by PKC*δ* siRNA during ischemia/reperfusion in cardiomyocytes [45]. In addition, PKC*δ* and Drp1 translocate to mitochondria membrane as a complex, which increase mitochondrial fission in Ang II or H_2_O_2_-injuried SH-SY5Y human neuroblastoma cells and in the rat model of hypertension-induced encephalopathy [21]. Consistent with previous studies, oxidative stress induced activation of PKC*δ*, which associated with and phosphorylated Drp1 in cultured neurons. PKC*δ* also translocated Drp1 from cytoplasm to mitochondria upon oxidative stress. The PKC*δ* inhibitor Rottlerin inhibited the interaction and translocation of PKC*δ* and Drp1 and the phosphorylation of Drp1, resulting in attenuated mitochondrial fission. These results suggest that oxidative stress-induced PKC*δ* activation impaired neuronal mitochondrial morphology, at least in part, by inducing Drp1-dependent fragmentation of the mitochondria. Thus, finding a medicine regulating PKC*δ*/Drp1 pathway mediated mitochondria function will be of particular importance in ischemic stroke. As an active compound of YQFM, ginsenoside Re protects methamphetamine-induced mitochondrial dysfunction and apoptosis through inhibition of PKC*δ* in dopaminergic cells in vivo and vitro [33, 55]. However, the effect of YQFM on PKC*δ* activation in ischemic stroke induced neuronal damage has not been fully elucidated. In our study, we provide the first evidence that both YQFM and Mdivi-1 inhibited PKC*δ* translocation to mitochondria and the interaction with Drp1 upon neuronal oxidative stress, which was similar with the effects of the PKC*δ* inhibitor Rottlerin. All these findings indicate that YQFM ameliorates oxidative stress-induced neuronal apoptosis through inhibiting PKC*δ*/Drp1 pathway-mediated mitochondrial fission.

The involvement of mitochondrial fission in cellular apoptosis or death has been demonstrated under different stimuli associated to stress [17]. It is reported that mitochondrial fission mediates high glucose-induced cell death through ROS production [56]. Besides, amyloid-beta oligomer induces neuronal cell death by inhibiting ERK/Drp1-mediated mitochondrial fragmentation [57]. Consistently, we demonstrated that cerebral ischemia and oxidative stress induced excessive mitochondria fission accompanied with apoptosis. YQFM prevented sequestration of antiapoptotic proteins Bcl-xl and Bcl-2, and activation of proapoptotic proteins Bax and Bak induced by ischemic stroke, suggesting its protection against mitochondrial apoptosis. Moreover, phosphorylation of Drp1 culminated after 6 h of H_2_O_2_ incubation and decreased thereafter (data not shown). Previous studies in our group demonstrated that H_2_O_2_ treatment for 12 h induced the executive phase of apoptosis in cultured neurons [58]. Mitochondrial fission and Drp1 activation occurred before caspase activation. Thus, mitochondrial fission is involved in the early apoptotic stage of neurons. YQFM inhibited mitochondria fission-mediated apoptosis induced by ischemic stroke and the related oxidative stress.

The morphology and function of mitochondria are determined by the balanced processes of fission and fusion. Because mitochondrial fusion has been reported to have antiapoptotic activity [59, 60], we cannot eliminate the possibility that oxidative stress-induced mitochondrial fission is linked with a disruption of fusion. Therefore, the regulation of YQFM on mitochondrial fusion and the fusion-related proteins deserves further investigation. Moreover, activation of PKC*δ* involves the translocation and phosphorylation in response to various stimuli [61]. As PKC*δ* is a key regulator of mitochondrial fission in CNS diseases [54], additional work is required to fully understand the activation of PKC*δ* under ischemic conditions and how YQFM regulates the action of PKC*δ*.

**Figure 7:**
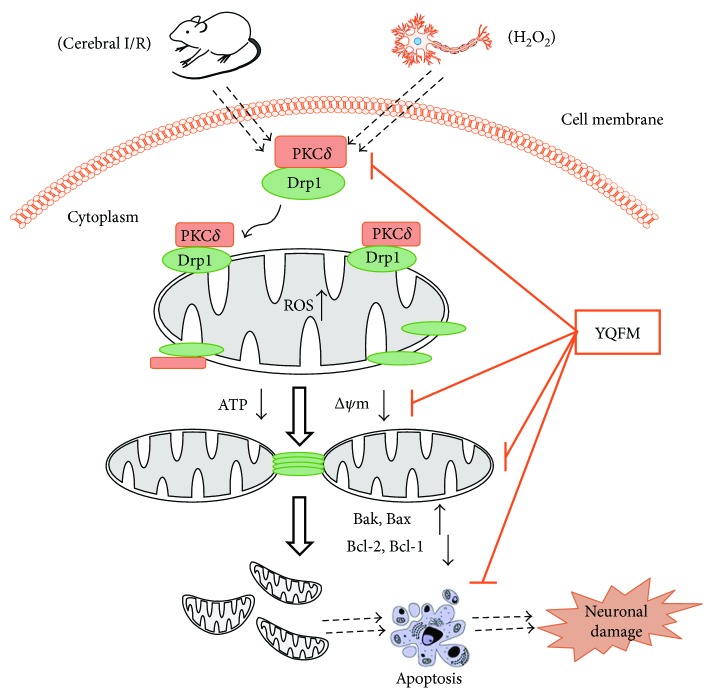
Schematic overview of mechanisms of YQFM on cerebral ischemia and oxidative stress induced neuronal mitochondrial fission and apoptosis. Cerebral ischemia or oxidative stress activates PKC*δ*, which associates with Drp1 in the cytosol. PKC*δ* activates, phosphorylates, and translocates Drp1 from cytosol to the outer mitochondrial membrane, leading to excessive mitochondrial fission and dysfunction, which are inhibited by YQFM treatment. In addition, YQFM attenuates oxidative stress-induced mitochondrial dysfunction and apoptosis through increasing ATP level, Δ*ψ*m, inhibiting ROS production, and regulating Bcl-2 family proteins levels.

Here, we demonstrated that YQFM, a compound injection, diminishes excessive mitochondrial fission through PKC*δ*/Drp1 signaling pathway, associated with inhibiting mitochondria apoptosis and dysfunction, which altogether contribute to the protection against ischemic stroke-induced neuronal injury (a possible mechanism shown in Figure 7). These findings elucidate a better understanding of the underlying molecular mechanisms of the increased mitochondrial fission-mediated neuronal apoptosis and provide an effective approach for the ischemic stroke therapy.

## Abbreviations

ANOVA: One-way analysis of variance
AraC: Cytosine *β*-D-arabinofuranoside
ATP: Adenosine 5′-triphosphate
BBB: Blood-brain barrier
BSA: Bovine serum albumin
CNS: Central nervous system
Co-IP: Coimmunoprecipitation
DAPI: 4′,6-Diamidino-2-phenylindole
DMSO: Dimethyl sulfoxide
Drp1: Dynamin-related protein 1
ECL: Enhanced chemiluminescence
FBS: Fetal bovine serum
H&E: Hematoxylin and eosin
H_2_O_2:_: Hydrogen peroxide
I/R: Ischemia/reperfusion
MTT: 3-(4,5-Dimethylthiazol-2-yl)-2,5-diphenyl tetrazoliumbromide
NAC: N-acetyl-L-cysteine
OD: Optical densities
PLL: Poly-L-lysin
PKC*δ:*: Protein kinase C-delta
PVDF: Polyvinylidene fluoride
ROS: Reactive oxygen species
YQFM: YiQiFuMai powder injection
SBTI: Soybean trypsin inhibitor
tMCAO: Transit middle cerebral artery occlusion
TMRE: Tetramethylrhodamine ethyl ester perchlorate
TTC: 2,3,5-Triphenyltetra-zolium chloride.
